# Apparent posterior cerebral artery territory perfusion asymmetry on arterial spin labeling MRI is a common non-pathologic finding in patients with a unilateral fetal posterior cerebral artery

**DOI:** 10.1007/s00234-021-02794-9

**Published:** 2021-08-30

**Authors:** Abraham Noorbakhsh, Nikdokht Farid, Divya S. Bolar

**Affiliations:** 1 Department of Radiology, University of California San Diego, La Jolla, CA USA; 2 Center for Functional Magnetic Resonance Imaging, University of California San Diego, La Jolla, CA USA

**Keywords:** Fetal posterior cerebral artery, Arterial spin labeling, Cerebral perfusion imaging, Magnetic resonance imaging

## Abstract

**Purpose:**

To determine the frequency of apparent posterior cerebral artery (PCA) territory asymmetry seen on arterial spin labeling (ASL) imaging in patients with a unilateral fetal PCA, but without underlying clinical or imaging pathology to suggest true hypoperfusion.

**Methods:**

A search of radiology reports from 1/2017 through 6/2020 was performed with the inclusion term "fetal PCA.” Eighty patients were included with unilateral fetal PCA confirmed on MRA or CTA, with brain MRI including ASL imaging, and without conventional imaging abnormality or clinical symptoms referable to the PCA territories. Cases were evaluated by two independent readers for visually apparent PCA perfusion asymmetries. ASL imaging consisted of pseudocontinuous ASL with 1.5 s labeling duration and 2 s post-labeling delay adapted from white paper recommendations.

**Results:**

Thirteen of 80 cases (16.2%) had apparent hypoperfusion in the PCA territory contralateral to the side of the fetal PCA. Agreement between readers was near perfect (97.5%, κ = 0.91). This finding was more common in patients who were older, scanned on a 3.0 T magnet, and who had non-visualization of the posterior communicating artery contralateral to the fetal PCA.

**Conclusion:**

Apparent PCA hypoperfusion on ASL is not uncommon in patients with a contralateral fetal PCA who have no clinical or conventional imaging findings to suggest true hypoperfusion. This phenomenon is likely due to differential blood velocities between the carotid and vertebral arteries that result in differential arterial transit times and labeling efficiency. It is important for radiologists to know that apparent hypoperfusion may arise from variant circle of Willis anatomy.

## Introduction

Arterial spin labeling (ASL) is a non-contrast MR imaging technique used to assess cerebral perfusion and has increasingly become a common component of routine clinical brain MRI protocols, particularly after the 2015 publication of consensus recommendations for clinical use, also known as the ASL white paper [1]. ASL has demonstrated utility in evaluating a variety of neurologic diseases, including cerebrovascular disease, CNS neoplasms, epilepsy, vascular anomalies, and neurodegenerative disorders [2–9]. Pseudocontinuous ASL (pCASL) is the most common variant of ASL imaging used in practice and recommended by the white paper for clinical applications [1]. With pCASL, a series of radiofrequency (RF) pulses are applied to a labeling plane in the upper neck to generate a magnetically labeled bolus within the cervical arteries [10]. A post-labeling delay (PLD) allows this labeled blood to arrive at the imaging plane, disseminate out of the macrovasculature, and accumulate into the microvasculature and parenchyma. Cerebral perfusion images are subsequently generated. pCASL, however, is prone to several artifacts that can result in erroneous perfusion estimates [11].

As ASL is increasingly being used in routine brain MRI protocols across many practices, much work is still needed to better understand ASL imaging findings and potential artifacts in the context of normal anatomic variants. At our institution, we have periodically encountered patients with the normal anatomic variant of a unilateral fetal posterior cerebral artery (PCA) who exhibit decreased perfusion signal in the contralateral PCA territory, but without underlying clinical or imaging pathology to suggest true hypoperfusion. This finding is thought to be related to the fetal PCA territory being predominantly supplied by the internal carotid artery (ICA), whereas the non-fetal PCA territory is predominantly supplied by the vertebral arteries (VAs). Since there is a velocity differential in blood flowing in the ICAs relative to the VAs, differences in pCASL arterial transit time and labeling efficiency may arise and contribute to asymmetric label delivery to PCA territories (particularly at literature suggested PLDs [1]).

If this asymmetry is erroneously interpreted to reflect underlying pathology, this may result in substantial clinical impact to the patient. While this phenomenon has been investigated quantitatively in the specific population of elderly hypertensive patients on clinical trials [12], it has not been studied in a general patient population undergoing routine brain MRIs. We hypothesize that this phenomenon is not an uncommon occurrence in clinical practice and aim to determine its frequency in patients scanned with pCASL as part of our institution’s routine brain MRI protocol.

## Methods

### Study population

**Fig. 1:**
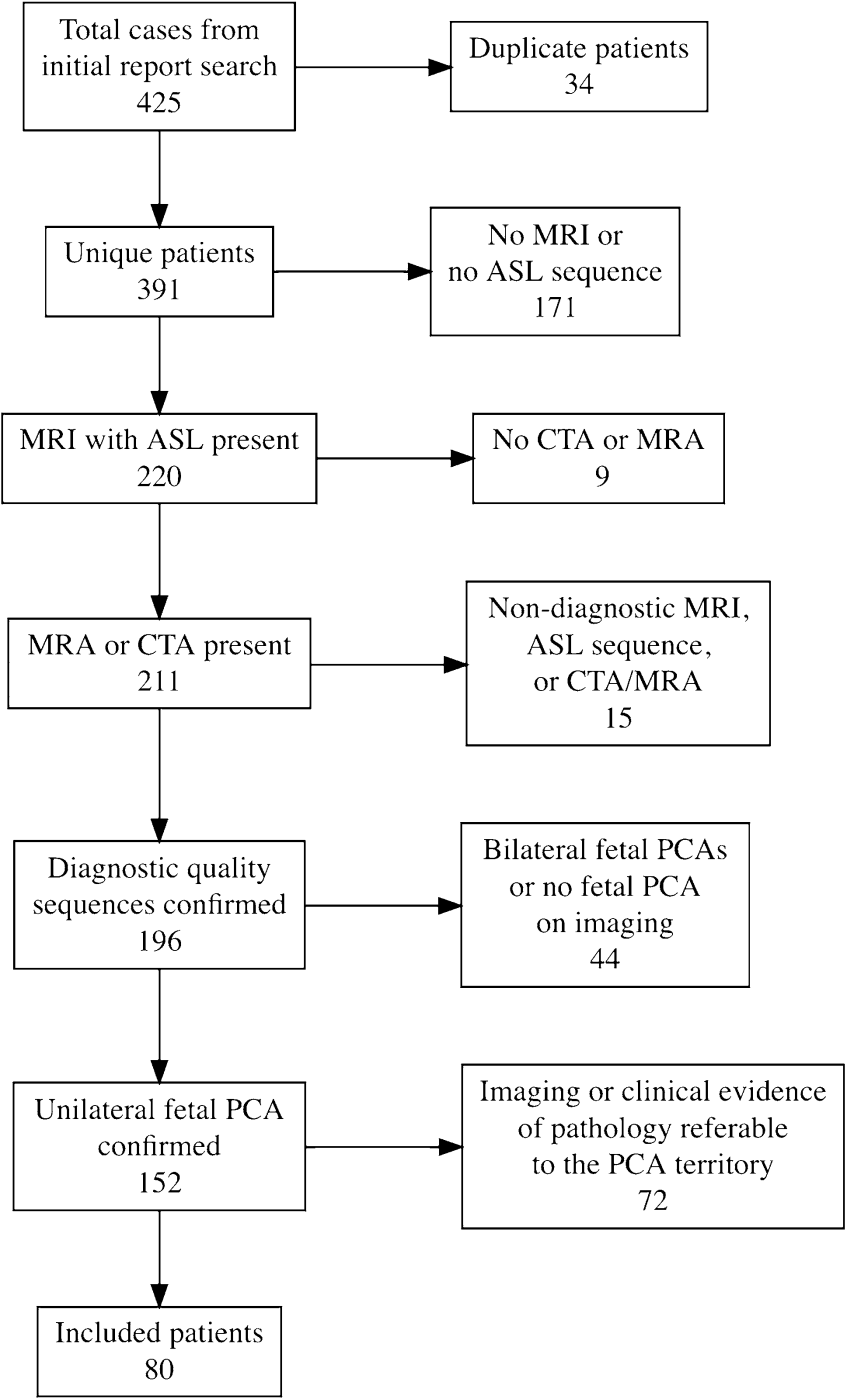
Flow chart demonstrating number of patients meeting inclusion and exclusion criteria

Starting in 2017, pCASL became incorporated into our institution’s routine brain MRI protocol. A text search of radiology reports at our institution from January 1, 2017, through June 30, 2020, was performed with the inclusion search term of “fetal PCA,” or some variant thereof. Searches were further qualified by requiring some variant of the terms “normal brain,” “unremarkable MRI,” or “no acute” in order to help narrow our search to patients without acute findings. Our initial search yielded a total of 425 MR and CT imaging reports for 391 unique patients. Each patient underwent a preliminary review to confirm the presence of a diagnostic quality brain MRI with inclusion of a pCASL sequence, an MRA or CTA of the brain confirming a unilateral fetal PCA, no evidence of a proximal arterial stenosis, and no clinical or conventional imaging findings referable to the PCA territory. Patients were excluded if clinical symptoms referable to the PCA territory were reported at the time of their MRI. These included homonymous hemianopia, quadrantanopia, hemisensory deficit, visual agnosia, prosopagnosia, hemineglect, and alexia without agraphia [13]. Conventional MRI sequences evaluated included DWI, FLAIR, and SWI images with special attention for the presence of acute or subacute infarct, acute hemorrhage, or increased oxygen extraction fraction. Of the 391 patients, 80 were ultimately included who met all the inclusion criteria and had no acute imaging findings (Fig. 1). There were no statistically significant differences in demographic characteristics (age and gender) between included and excluded patients. Although not included in the main cohort of interest, 18 of the 44 patients with bilateral fetal PCAs met the same exclusion criteria and were also evaluated as a control group. Approval from our institutional review board was obtained for this retrospective study.

### Imaging methods

Brain MRIs were obtained on either 1.5 T or 3 T MRI scanners. Conventional brain MRI imaging at our institution includes T1-weighted axial and sagittal, T2-weighted axial and coronal, FLAIR axial, diffusion weighted, susceptibility weighted, and ASL sequences. An abbreviated stroke protocol is occasionally performed, which includes diffusion weighted, FLAIR axial, susceptibility weighted, and ASL sequences. MRA and CTA studies used standard product angiography protocols.

ASL was performed using the GE product pCASL sequence (General Electric, Milwaukee, WI), which incorporates a 3D stack-of-spirals fast spin echo readout. pCASL-specific parameters include a labeling duration of 1.5 s and PLD of 2 s (adapted from ASL white paper recommendations), which were identical for both 1.5 T and 3.0 T scanners [1]. 3D spiral readout parameters include spiral interleaves = 8, points per spiral = 512, slices = 36, in-plane resolution: 3.64–4.53 mm^2^, slice thickness = 4.0–4.2 mm, FOV = 24–26 cm, TE = 9.5–10.5 ms, bandwidth = 62.5 kHz, TR = 4800–4847 ms, NEX = 3, and scan time = 4 m 32 s to 4 m 42 s.

### Image interpretation

Initial review of brain MRIs and MRA/CTA images was performed by a senior radiology resident (A.N.) to evaluate for the inclusion/exclusion criteria described above. Presence of a fetal PCA was confirmed on non-contrast axial 3D TOF MRA or contrast-enhanced CTA images, as defined by a robust PCOM with the ipsilateral P1 segment of the PCA either non-visualized or of smaller caliber than the PCOM. Among patients with confirmed unilateral fetal PCA, the ipsilateral P1 segment and contralateral PCOM were evaluated and further subclassified as either visualized or non-visualized, respectively, to determine if these subclassifications specifically associate with PCA perfusion asymmetry. All included patients were also confirmed to be free of proximal arterial stenoses via MRA or CTA images.

ASL images of the included patients were then independently reviewed in a blinded fashion by the senior radiology resident (A.N.) with 4 years of experience and attending neuroradiologist (D.S.B.) with 9 years of experience using a clinical workstation. Calibrated CBF perfusion maps were reviewed for any evidence of qualitative asymmetry in the ASL signal.

To provide a quantitative estimate of what was deemed visually apparent asymmetry, a circle region-of-interest (ROI) was placed in the posteromedial gray matter of each PCA territory at the level of the thalami. The mean and standard deviation of the ROI perfusion values (in ml/100 g-min) were recorded. The difference in perfusion between the PCA territories was defined by subtracting the mean from the side ipsilateral to the fetal PCA from the contralateral PCA territory. In patients with bilateral fetal PCAs, the absolute difference was obtained.

### Statistical analysis

Comparisons of differences in demographic and clinical/imaging variables were performed using Fisher’s exact test for categorical variables and *t*-test for continuous variables. Multivariate logistic regression analysis was performed using Firth’s bias reduction to address the relatively small sample size and potential for quasi-complete separation [14, 15]. The association of age, gender, ipsilateral P1 segment visualization, contralateral PCOM visualization, and MRI magnetic field strength (3.0 T vs 1.5 T) were evaluated in their association with the presence of pCASL-CBF perfusion asymmetry in the PCA territories on adjusted multivariate analysis. Firth’s logistic regression was implemented using the “logistf” package in R [16]. Inter-reader agreement was assessed using Cohen’s kappa coefficient with the “vcd” package [17]. All statistical analyses were performed using R, version 4.0.2 [18].

## Results

### Patient characteristics

The included 80 patients with a unilateral fetal PCA ranged in age from 23 to 93 years old with mean age of 60. There was a slight female predominance with 53 (66.2%) female to 27 (33.8%) male cases included. Demographic and imaging characteristics of the study population overall, as well as stratified by pCASL CBF perfusion asymmetry in the PCA territories, are summarized in Table 1.

**Table 1:** Demographic and imaging characteristics of the overall study population, as well as stratified by the presence of asymmetry on pCASL CBF perfusion imaging

Variables	Categories	Overall	No CBF asymmetry	CBF asymmetry	*p*-value
Number of patients, *n* (%)		80	67 (83.8)	13 (16.2)	
Age, mean ± SD		60 ± 15	58 ± 15	68 ± 14	0.002*
Age categories, *n* (%)	18–54	27	25 (92.6)	2 (7.4)	0.083
	55–64	21	17 (81.0)	4 (19.0)	
	65–74	15	14 (93.3)	1 (6.7)	
	75 +	17	11 (64.7)	6 (35.3)	
Gender, *n* (%)	Female	53	46 (86.8)	7 (13.2)	0.345
	Male	27	21 (77.8)	6 (22.2)	
Fetal PCA side, *n* (%)	Left	36	29 (80.6)	7 (19.4)	1
	Right	44	38 (86.4)	6 (13.6)	
Ipsilateral P1 PCA, *n* (%)	Visualized	39	33 (84.6)	6 (15.4)	0.551
	Non-visualized	41	34 (82.9)	8 (17.1)	
Contralateral PCOM, *n* (%)	Visualized	42	39 (92.9)	3 (7.1)	0.032*
	Non-visualized	38	28 (73.7)	10 (26.3)	
MRI Field Strength, *n* (%)	1.5 Tesla	51	49 (96.1)	2 (3.9)	< 0.001*
	3.0 Tesla	29	18 (62.1)	11 (37.9)	
ΔCBF_PCA_^a^, mean ± SD		4.7 ± 9.6	0.9 ± 3.2	24.4 ± 6.8	< 0.001*

^*^Denotes significance at the *p* < 0.05 level

^a^ΔCBF_PCA_ was defined as the perfusion signal from the ipsilateral PCA territory subtracted from the contralateral PCA territory, units in mL/100 mg/min

### Visually apparent perfusion asymmetry

**Fig. 2:**
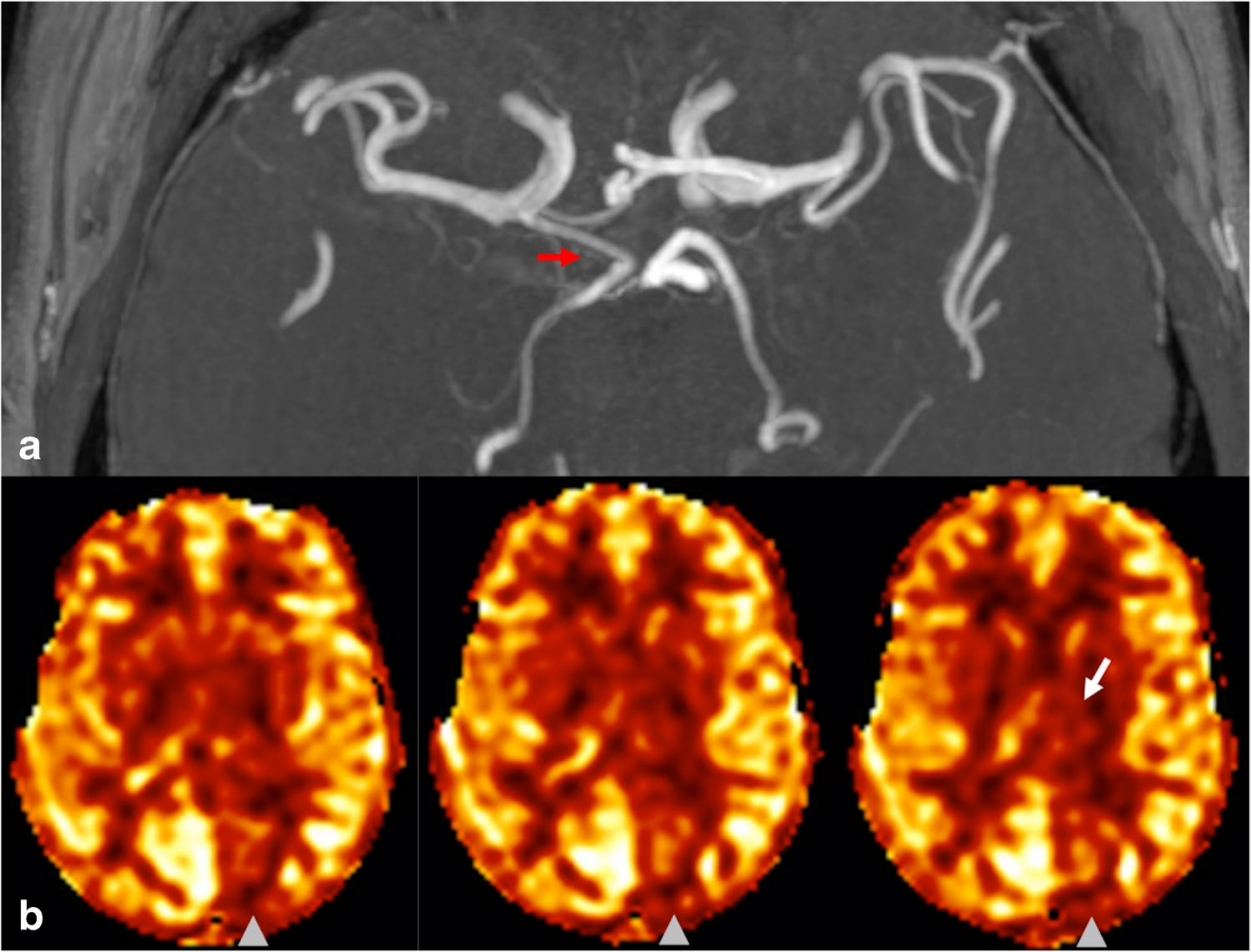
Case of a women in her sixties presenting for work-up of transient lightheadedness and blurred vision that self-resolved, attributed to non-ischemic factors. **a** MIP MRA image of the Circle of Willis demonstrates a right fetal PCA (red arrow) without a visible contralateral PCOM. **b** Three consecutive calibrated pCASL CBF perfusion images demonstrate decreased perfusion in the left PCA territory (arrowhead) including the left thalamus (white arrow), both contralateral to the right fetal PCA

**Fig. 3:**
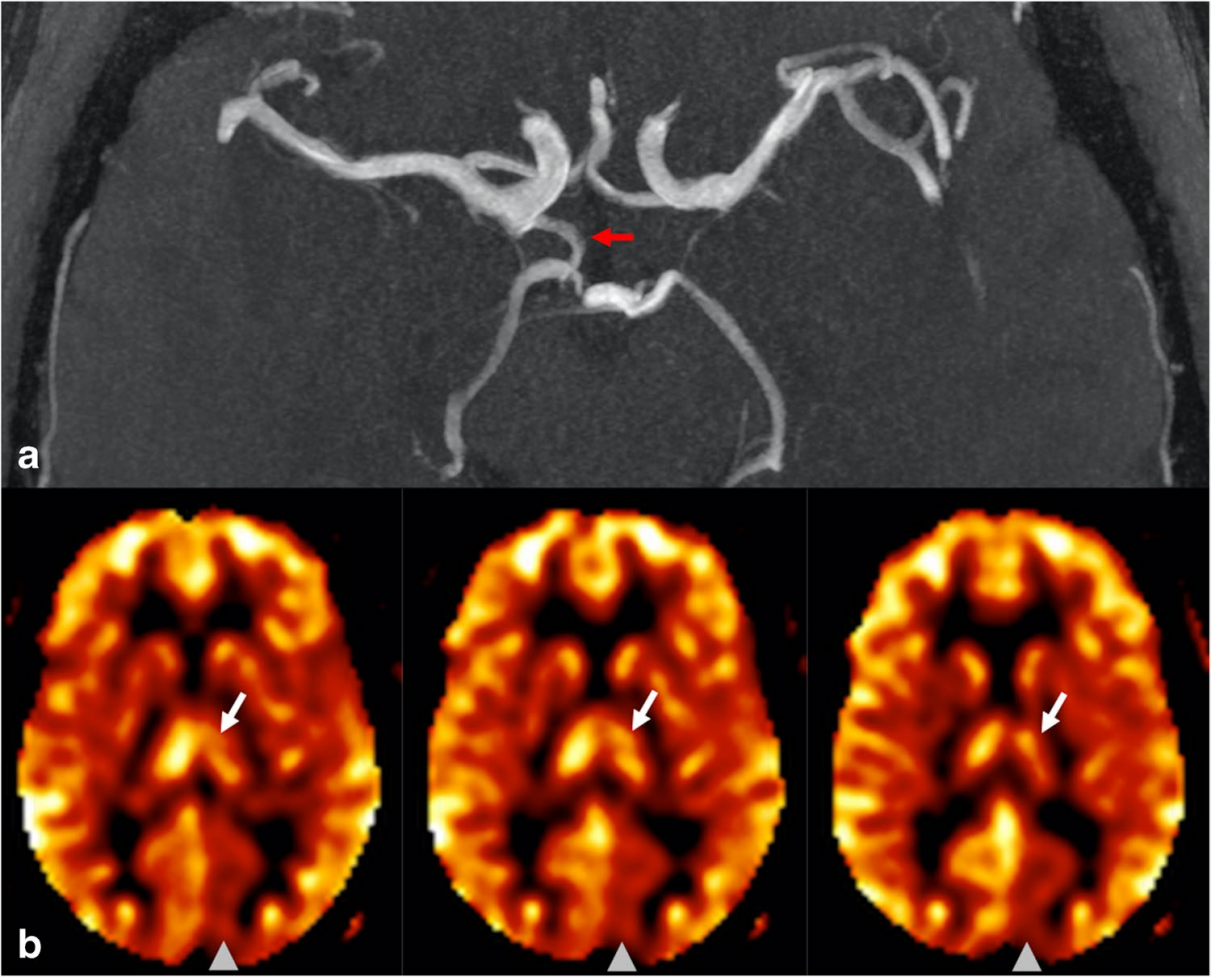
Case of a woman in her late seventies presenting for follow-up of a 5-mm right PCOM aneurysm. **a** MIP MRA image of the Circle of Willis demonstrates a right fetal PCA (red arrow). The aneurysm is not well visualized on MIP images, but was unchanged in size when evaluated on thin slice images (not shown). **b** Three consecutive calibrated pCASL CBF perfusion images demonstrate decreased perfusion in the left PCA territory (arrowhead) including the left thalamus (white arrow), both contralateral to the right fetal PCA

**Fig. 4:**
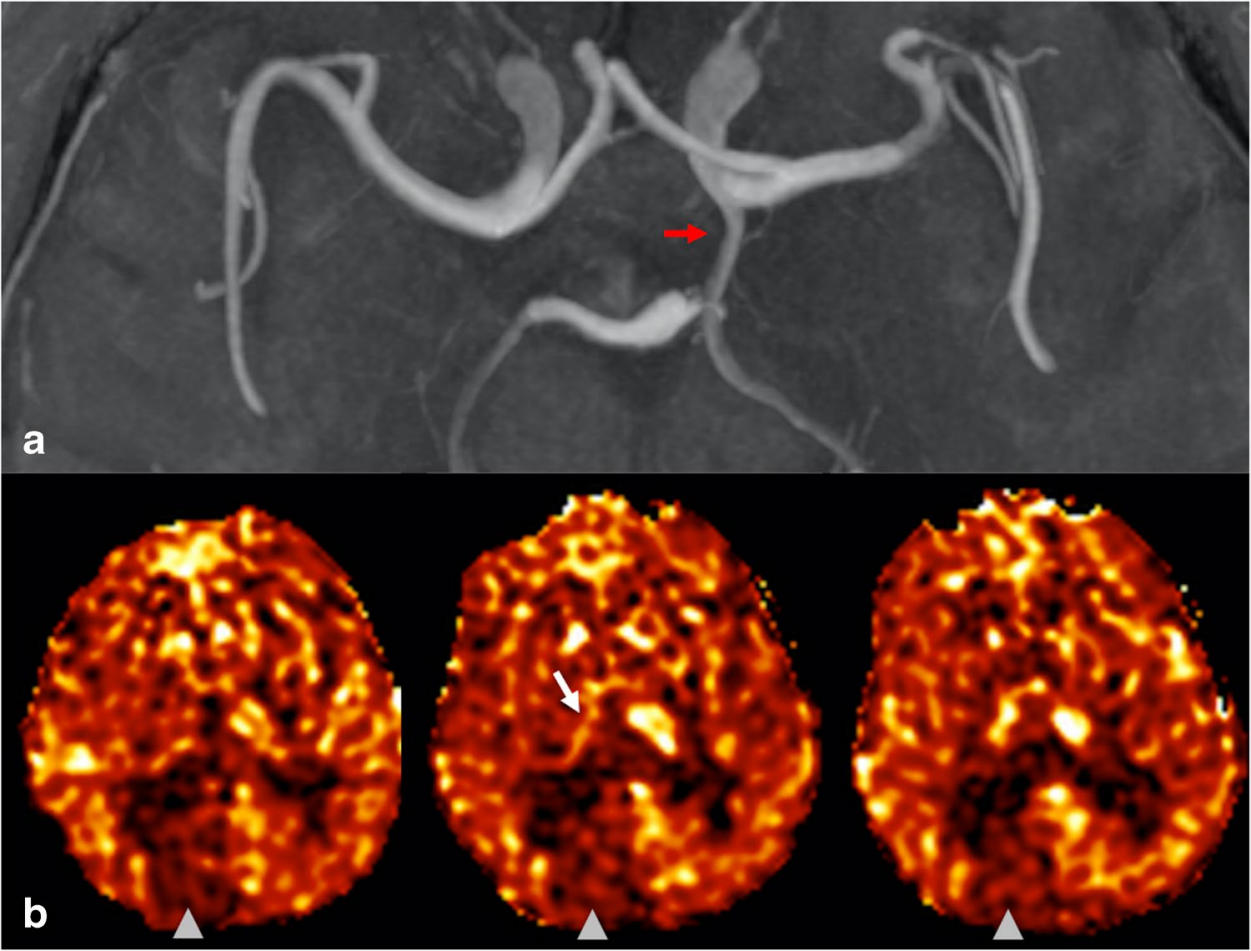
Case of a man in his late seventies presenting with paroxysmal vertigo for 2 weeks. MRA imaging demonstrates a distal left PICA stenosis (not shown), thought contributory to his symptoms. **a** MIP MRA image of the Circle of Willis demonstrates a left fetal PCA (red arrow). **b** Three consecutive calibrated pCASL CBF perfusion images demonstrate decreased perfusion in the right PCA territory (arrowhead) including the right thalamus (white arrow), both contralateral to the left fetal PCA

Both readers (A.N. and D.S.B.) identified 13 cases out of 80 (16.2%) that had apparent decreased perfusion signal in the PCA territory contralateral to the side of the fetal PCA. Agreement between readers was near-perfect with an inter-reader agreement of 97.5% and Cohen’s kappa of 0.91 (95% confidence interval 0.78–1.00). The two cases with disagreement were reviewed and discussed by the readers together to reach a final consensus on the presence of pCASL CBF perfusion asymmetry. In all 13 cases with CBF asymmetry, the PCA territory perfusion signal ipsilateral to the fetal PCA qualitatively matched the perfusion signal of the ipsilateral anterior circulation (and did not demonstrate relative hypo- or hyperperfusion), while the contralateral PCA territory appeared hypoperfused relative to the other major vascular territories. None of the 13 cases showed PCA territory infarction on DWI/FLAIR sequences, increased oxygen extraction on SWI, or focal neurological symptoms referable to the PCA territory that would suggest a true perfusion abnormality. Three example cases of apparent PCA territory hypoperfusion contralateral to a unilateral fetal PCA are shown in Figs. 2, 3 and 4.

### Predictors of PCA territory pCASL CBF Asymmetry

Patients with apparent hypoperfusion in the PCA territory contralateral to the side of the fetal PCA were older (mean age 68 vs 58 without asymmetry, *p* = 0.039), were more likely to have been scanned on a 3.0 T magnet (37.9% of patients scanned on a 3.0 T showed the asymmetry versus 3.9% of patients scanned on a 1.5 T, *p* < 0.001), and were more likely to have a non-visualized contralateral PCOM (26.3% of patients with a non-visualized contralateral PCOM showed the asymmetry vs. 7.1% with a visualized contralateral PCOM, *p* = 0.032). These associations continued to hold true on multivariate logistic regression analysis with Firth’s bias reduction after adjusting for other demographic and imaging characteristics (see Table 2).

**Table 2:** Multivariate Firth’s logistic regression for visually apparent PCA territory pCASL-CBF perfusion asymmetry

Variables	Categories	Adjusted OR (95% CI)	*p*-value
Age (range 23–93)	Continuous	1.06 (1.01–1.12)	0.021*
Gender	Male	3.55 (0.79–20.91)	0.101
	Female	REF	
Fetal PCA side	Right	0.94 (0.22–4.08)	0.928
	Left	REF	
Ipsilateral P1 PCA	Non-visualized	0.79 (0.18–3.41)	0.755
	Visualized	REF	
Contralateral PCOM	Non-visualized	5.74 (1.30–35.16)	0.02*
	Visualized	REF	
MRI field strength	3.0 Tesla	23.45 (4.68–211.64)	< 0.001*
	1.5 Tesla	REF	

^*^Denotes significance at the *p* < 0.05 level

### Quantitative estimates of pCASL CBF asymmetry

Among patients with visually apparent perfusion asymmetry, the mean difference in perfusion signal (ΔCBF_PCA_) was 24.4 ± 6.8 mL/100 g/min, consistently higher in the contralateral PCA territory relative to the ipsilateral PCA territory. This difference was statistically significantly relative to patients without visualized asymmetry (at *p* < 0.001, noting ΔCBF_PCA_ = 0.9 ± 3.2 mL/100 g/min in patients without asymmetry).

Although not included in the main cohort of interest for this study, 18 patients with bilateral fetal PCAs were also assessed as a control group, none of which showed qualitative or quantitative pCASL CBF asymmetries (ΔCBF_PCA_ = 0.3 ± 1.2 mL/100 g/min).

## Discussion

Apparent PCA territory hypoperfusion on pCASL imaging is not uncommon in patients with a unilateral (contralateral) fetal PCA. This almost certainly does not reflect true hypoperfusion, given lack of clinical or conventional imaging findings referable to the territory. Although Wolf et al. previously characterized quantitative differences in pCASL-derived CBF among patients with a unilateral fetal PCA [12], this paper builds on that work by (1) characterizing the frequency of visually apparent PCA territory perfusion asymmetry in unilateral fetal PCA patients encountered in routine clinical practice; (2) investigating several clinical and imaging parameters associated with this phenomenon, such as older age, non-visualization of a contralateral PCOM, and scanning at 3.0 T magnetic field strength; and (3) doubling the sample size from 40 to 80 unilateral fetal PCA patients and including all-comers as opposed to elderly hypertensive patients on clinical trial. A similar investigation has been done with dynamic susceptibility contrast (DSC) perfusion imaging, but due to fundamental differences in the physics and bolus dynamics between DSC and ASL, different conclusions were reached [19].

The etiology of this apparent hypoperfusion is felt to be related to lower blood velocity in the VAs supplying the non-fetal PCA territory relative to ICAs supplying the fetal PCA territory. This velocity difference would result in (1) a longer arterial transit time (ATT) from the pCASL labeling plane to the tissue supplied by the VAs and (2) decreased labeling efficiency in the VAs [10, 12]. Thus, fewer labeled spins are delivered to the non-fetal PCA territory during the 2 s PLD, resulting in the apparent hypoperfusion. Since the fetal PCA territory is supplied by the ICA, it has a similar qualitative appearance to the anterior circulation territories, accentuating the apparent hypoperfusion in the non-fetal PCA territory. Of note, lower velocities in the VAs relative to the ICAs have been reported previously on phase-contrast MRI and sonography [20, 21].

Patients with a non-visualized contralateral PCOM are more likely to exhibit the asymmetry since the associated PCA territory will receive supply almost exclusively from the VAs. In contrast, a robust contralateral PCOM will result in mixed anterior and posterior circulation supply, diminishing the left–right PCA asymmetry. Non-visualization of the ipsilateral P1 PCA did not associate with PCA perfusion asymmetry, likely since a fetal PCA with a visualized P1 is more typically reported (and thus included in the study) if the P1 is diminutive, in which case only minimal supply comes from the posterior circulation (similar to as if it were not visualized).

Our findings are supported by the previously mentioned study from Wolf et al., who also contend that perfusion asymmetry seen in patients with a unilateral fetal PCA are mediated by differential velocities in the supplying arteries [12]. Our finding that patients scanned at 3 T were more likely to exhibit the artifact than patients scanned at 1.5 T supports differential labeling efficiency as a mechanism, which is likely exacerbated at 3 T due to more pronounced magnetic field inhomogeneities [22–25].

We also identified that the diminished PCA territory perfusion signal contralateral to a fetal PCA was more common in older patients. A study by Scheel et al. evaluates the effect of age on flow velocity in the cervical arteries and reports a larger decrease in the VAs relative to the ICAs (approximately 29% decrease in the VAs in the 60–85 age group relative to the 29–39 age group, compared to a 19% decrease in the ICAs) [26]. This larger reduction in the vertebral artery velocity with age may result in further exacerbation of ATT and labeling efficiency effects and may, in-part, explain why the artifact is seen more frequently among older patients.

Of note, the majority of patients with fetal PCA configurations did not appear to demonstrate PCA perfusion symmetry. This may be be due to a smaller differential velocity between the ICAs and VAs (e.g., in younger populations) that results in (1) similar ATTs for both arteries for which a PLD of 2 s is sufficient for complete label delivery, and (2) negligible difference in labeling efficiency.

A potential limitation of this study is that we did not directly control for the angle of the labeling plane, which could also result in asymmetric labeling of vessels. However, clinical pCASL sequences do not routinely incorporate manual specification of the labeling plane. Another limitation is that we did not directly measure the flow velocity of the supplying vessels to further investigate the differential arterial velocities as a mediator of this phenomenon. A third limitation is that our gray matter ROIs for CBF will unintentionally include white matter voxels, which will introduce slight error into the quantitative analysis. Finally, the retrospective nature of the study confines us to reviewing routine MR images already acquired. A prospective study would enable us to alter imaging parameters to determine if the phenomenon may be mitigated, for example, by (1) employing a multi-PLD approach that controls for arterial arrival times, (2) increasing the PLD to allow more complete label delivery to the PCA territory, or (3) employing velocity-selective ASL approaches that are insensitive to arterial transit times [27].

## Conclusion

The normal anatomic variant of a unilateral fetal PCA can result in apparent pCASL CBF hypoperfusion in the contralateral PCA territory among patients scanned during routine clinical practice (up to 16% in this study). This phenomenon is more common among patients scanned on 3.0 T MRIs, patients with a non-visualized PCOM contralateral to their fetal PCA, and older patients. Radiologists should be aware that apparent hypoperfusion may arise from variant circle of Willis anatomy, which should not be incorrectly interpreted as true hypoperfusion. The use of longer PLD times and/or velocity-based ASL approaches may correct this asymmetry and is an area of future research. As ASL imaging becomes increasingly routine in standard brain MRI protocols in many practices, it is important to be aware of potential imaging findings that result from normal anatomical variation rather than underlying pathology.

## Data Availability

Data is currently not available, but descriptive data that support the findings of the study could be made available from the corresponding author on reasonable request.

## Code availability

R code is available upon request.
